# High-dimensional single photon based quantum secure direct communication using time and phase mode degrees

**DOI:** 10.1038/s41598-024-51212-6

**Published:** 2024-01-09

**Authors:** Byungkyu Ahn, Jooyoun Park, Jonghyun Lee, Sangrim Lee

**Affiliations:** Communication and Media Standard Lab., LG Electronics, Seoul, 06772 South Korea

**Keywords:** Engineering, Electrical and electronic engineering, Optical physics, Quantum optics, Single photons and quantum effects, Optics and photonics, Fibre optics and optical communications, Physics, Quantum physics, Quantum information, Qubits, Single photons and quantum effects

## Abstract

Quantum secure direct communication (QSDC) can guarantee security using the characteristics of quantum mechanics even when a message is directly transmitted through a quantum channel without using a secret key. However, the transmission rate of the QSDC is limited by the dead time of a single photon detector (SPD) as well as channel loss over the distance. To overcome this limited transmission rate, we propose a high-dimensional single photon-based QSDC protocol that applies two optical degrees of freedom: time and phase state. First, an *N*-dimensional time and phase state generation method that considers the dead time is proposed to minimize the measurement loss of a transmitted message. Second, among the two types of quantum states, the phase state with relatively low measurement efficiency is used only for eavesdropping detection, and the time state is used for sending messages with differential delay time bin-based encoding techniques. Lastly, we propose an efficient method for measuring *N*-dimensional time and phase-based quantum states and recovering classical bit information. This study performs security analysis against various attacks, and verifies the transmission rate improvement effect through simulation. The result indicates that our proposal can guarantee higher security and transmission rates compared to the conventional DL04 QSDC.

## Introduction

Quantum secure communication is a method that provides unconditional security of information exchanged between a transmitter and a receiver using a law of quantum mechanics. Thus, quantum secure communication can achieve absolute security from threats where current security techniques can be broken, such as Rivest–Shamir–Adleman (RSA) algorthm^1^ that is based on computational hardness assumption, owing to the advent of an eavesdropper having powerful computing abilities including quantum computers^2^. Currently, different types of quantum secure communication methods are being researched, and representative protocols include quantum key distribution (QKD)^3–8^, quantum secret sharing (QSS)^9,10^, and quantum secure direct communication (QSDC)^11–15^.

QKD is a quantum cryptography technology proposed by Benett and Brassard^5^ in the 1980s. It is the most widely researched quantum secure communication protocol that protects a symmetric secret key from an eavesdropper’s attack to be safely shared among users through a quantum channel. In QKD, the information security is guaranteed by verifying the existence of an eavesdropper through the monitoring of a part of information transmitted through a quantum channel. The symmetric key, which is shared between a transmitter and a receiver through a quantum channel, is used to encrypt plaintext into ciphertext at the transmitter, and then the ciphertext is sent to the receiver via a public channel, and decrypted using the identical key at the receiver to recover the plaintext.

The basic concept of QSDC was introduced in 2000 by Long and Liu^11^ and can be broadly classified as an entanglement-based 2-step protocol^16^ and a single photon-based DL04 protocol^17^. In the two decades since QSDC was first developed, it has been actively studied in both theoretical^18–29^ and experimental^30–33^ domains. Unlike QKD, QSDC is a protocol that does not separate the transmission process of a message and a secret key, and the secret message is directly sent through a quantum channel without applying a secret key. Furthermore, QSDC guarantees security by applying the identical quantum phenomena such as the no-cloning theorem and uncertainty principle as QKD. Accordingly, QSDC has several advantages over QKD. Firstly, QSDC does not need to consider the problems related to management and storage of large amounts of secret keys, which becomes a burden as the number of users increases in QKD. Secondly, QSDC sends a message after verifying whether an eavesdropper has attempted an attack. Thus, even if an eavesdropper attempts an attack, the information leakage related to the message can be prevented. Third, because the encryption and decryption of the secret key do not need to be conducted, the overall system complexity can be reduced, and the occurrence of information leakage which may occur in these processes can be avoided as well.

However, the transmission rate, which is too low compared to the transmission rate of conventional digital communication, is a significant obstacle for QSDC to be practically used as a data transmission technology. The major reason for the low transmission rate of QSDC is the dead time^34^ of a single photon detector (SPD) used during the measurement process. The dead time represents the reset time between the first occurrence of the detection event and the subsequent detection event in the detector; if the following signal is received within the reset time, the detector cannot detect the signal. Even the superconducting nanowire single photon detector(SNSPD), which is currently known to have the shortest dead time requires at least 10 ns of dead time^35^. Therefore, the transmission rate of QSDC is inevitably limited by the SPD dead time (see supplementary information).

In the current situation where hardware limitations exist, the most effective method for increasing the transmission rate without physically improving the SPD’s dead time is using a high dimensional encoding method. When this method is applied, $\lfloor \log _{2} N \rfloor$ bits of information per qubit can be transmitted using $\textit{N}$-dimensional encoding^36–38^ on the quantum communication protocol. For the high dimensional encoding technique, many researches are being conducted on different methods involving various optical degree of freedom (ODOF) including temporal^39,40^, spatial^41–43^, and momentum^44,45^ methods. While the single photon based QSDC protocol is also being studied using various ODOF^46–48^, the transmission information is still 1 bit per qubit and achieving a high transmission rate of the QSDC is extremely difficult, considering the commercial SPD dead time.

To improve this issue, we propose an efficient $\textit{N}$-dimensional QSDC system that can increase the transmission rate by applying a high dimensional encoding technique based on two types of ODOF, time and phase states, on the single photon based DL04 QSDC protocol. The main contributions of this study are as follows. Firstly, to minimize the information loss occurring in the measurement process, the phase state, which is one of the two ODOF, is used for the quantum bit error rate (QBER) estimation to determine the existence of eavesdropper in the quantum channel. The phase state is not used for message transmission because as the phase state dimension is increased by a factor of two, the measurement probability using the time delay interferometer is decreased by a factor of two, which causes the information loss to increase by the same factor. On the other hand, the time state is used for two purposes: QBER estimation and message transmission. However, even in the time state, if the interval between the previous signal and current signal is shorter than the SPD dead time, the information loss occurs because the detector does not detect the current signal. In our proposal, guard time is added between time states to prevent the information loss due to dead time. Secondly, in the single photon based QSDC protocol, the transmitter must generate an encoded time state by message coding on the initial time state received from the receiver. In this process, the transmitter must perform message encoding without measuring the initial time state. In order to generate an encoded time state without knowing the initial time state, we add a time delay corresponding to the classical message to the initial time state. Thirdly, we introduce a method for measuring *N*-dimension optical quantum states when the QSDC is configured by expanding the time and phase states to *N* dimensions, and analyze the measurement efficiency and measurement methods of time and phase quantum states according to dimensional changes. Finally, we demonstrate through a simulation that the transmission rate can be improved by the proposed *N*-dimensional QSDC protocol compared to the classical two-dimensional DL04 protocol at the same transmission distance. Additionally, to verify the security of the proposed QSDC, we consider the following two types of attack methods. First, security from intercept resend attack is guaranteed from having a higher QBER when compared to conventional two-dimensional QSDC. Next, the security is proved by improving the secrecy capacity obtained by wiretap channel theory compared to the conventional two-dimensional QSDC.

## Results

### *N*-dimensional mutually unbiased bases using the time and phase state

The general meaning that the two bases in the *N*-dimension are mutually unbiased bases (MUBs)^49^ is as follows. One of the MUBs is known as the computational basis,

1 $$\begin{aligned} MUB_C=\{|A_0\rangle , |A_1\rangle , |A_2\rangle , ... , |A_{N-1}\rangle \}. \end{aligned}$$

The other basis is generated by applying the discrete quantum Fourier transform to the computational basis and is called the dual basis,

2 $$\begin{aligned} MUB_D=\{|A_0^{'}\rangle , |A_1^{'}\rangle , |A_2^{'}\rangle , ... , |A_{N-1}^{'}\rangle \}, \end{aligned}$$

3 $$\begin{aligned} |A_k^{'}\rangle =\frac{1}{\sqrt{N}}\sum _{j=0}^{N-1}\ W_N^{jk}|A_j\rangle \ {,} \quad W_N=\exp {i\frac{2\pi }{N}}. \end{aligned}$$

If the squares of magnitude of the inner product between any two basis states $|A_l\rangle$ and $|A_k^{'}\rangle$ are equal to the reciprocal of the dimension *N*, the two orthogonal bases $MUB_C$ and $MUB_D$ are mutually unbiased in *N* dimensional Hilbert space $H^N$.

4 $$\begin{aligned} |\langle A_l|A_k^{'}\rangle |^{2}=\frac{1}{N}{.} \end{aligned}$$

Equation (4) means that when a state corresponding to one of the two bases is prepared, the results of all measurements occur with the same probability from a different basis point of view.

In this paper, we apply these two MUBs to time and phase based high-dimensional encoding. First, we apply the time basis states $|t_k\rangle$ $( k = 0, \ldots ,N-1)$ as a computational basis. In addition, the phase basis states $|p_k\rangle$ are generated as dual basis through the discrete quantum Fourier transform operation in the time state $|t_k\rangle$ .

5 $$\begin{aligned} |p_{k}\rangle =\frac{1}{\sqrt{N}}\sum _{j=0}^{N-1}\ \exp {\left( \frac{2ijk\pi }{N}\right) }|t_{j}\rangle \ {,} \quad where \quad k=0,\ldots , N-1. \end{aligned}$$

**Figure 1:**
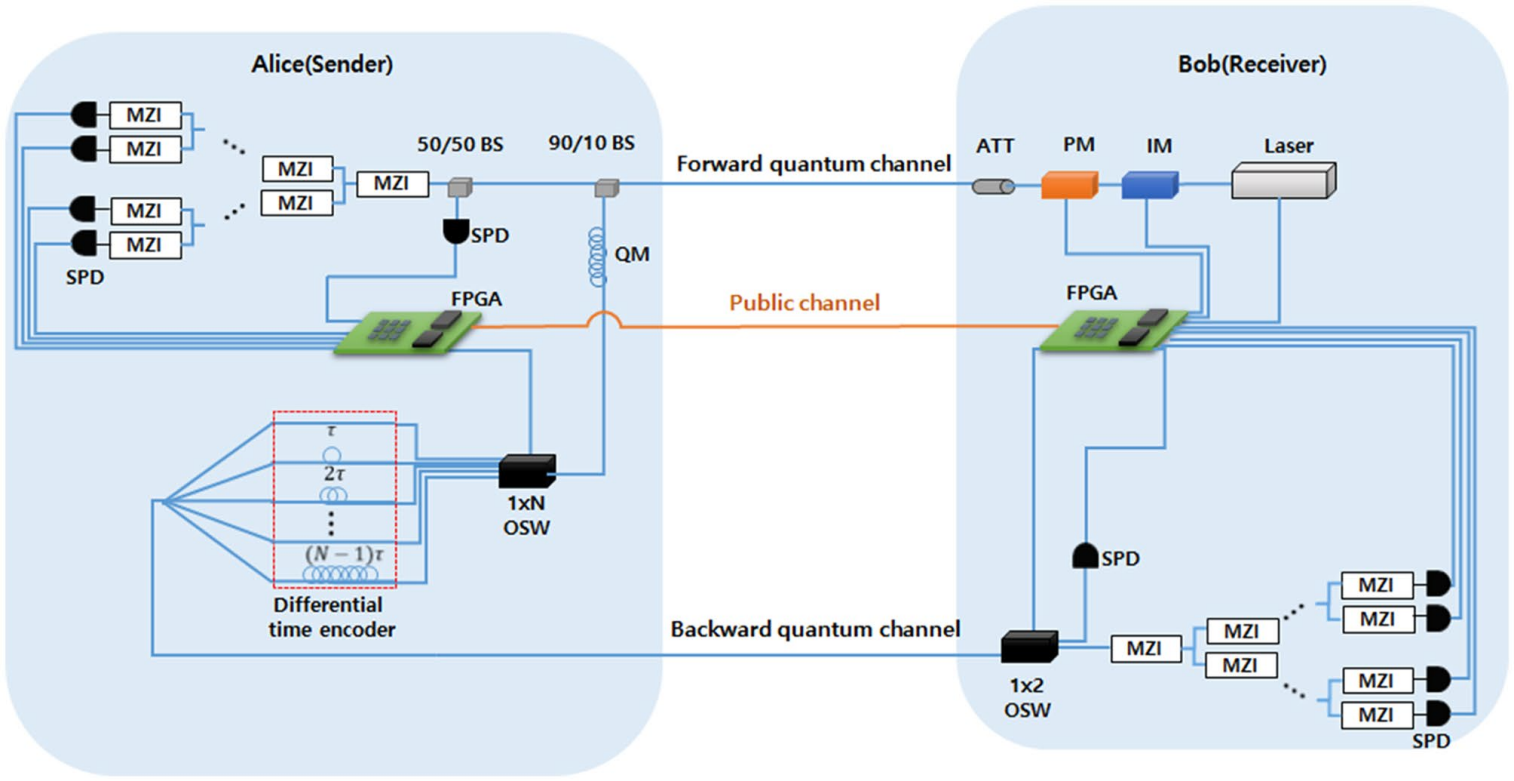
The diagram of proposed *N*-dimensional QSDC protocol. IM denotes the intensity modulator. PM is phase modulator. An attenuator (ATT) reduces the photon level. BS denotes the beam splitter. QM is quantum memory or storage line. Optical switch (OSW) allows that the photon from different paths to be transmitted to the desired paths. When measuring the phase state, Mach–Zehnder interferometer (MZI) is used. The entire QSDC system is controlled by the field programmable gate array (FPGA).

### *N*-dimensional QSDC protocol description

In this section, we propose a high dimensional QSDC scheme as shown in Fig. 1 to overcome the limited transmission rate of the QSDC protocol caused by the saturation of the detection rate of SPD. The protocol presents an efficient way of securely transmitting messages by constructing non-orthogonal and mutually unbiased basis states using two types of ODOF, time and phase states. Based on the two ODOFs, proposed *N*-dimensional QSDC protocol can be described as the following steps:

#### Initial *N*-dimensional time and phase state preparation

Bob Generates initial time and phase state.

**Figure 2:**
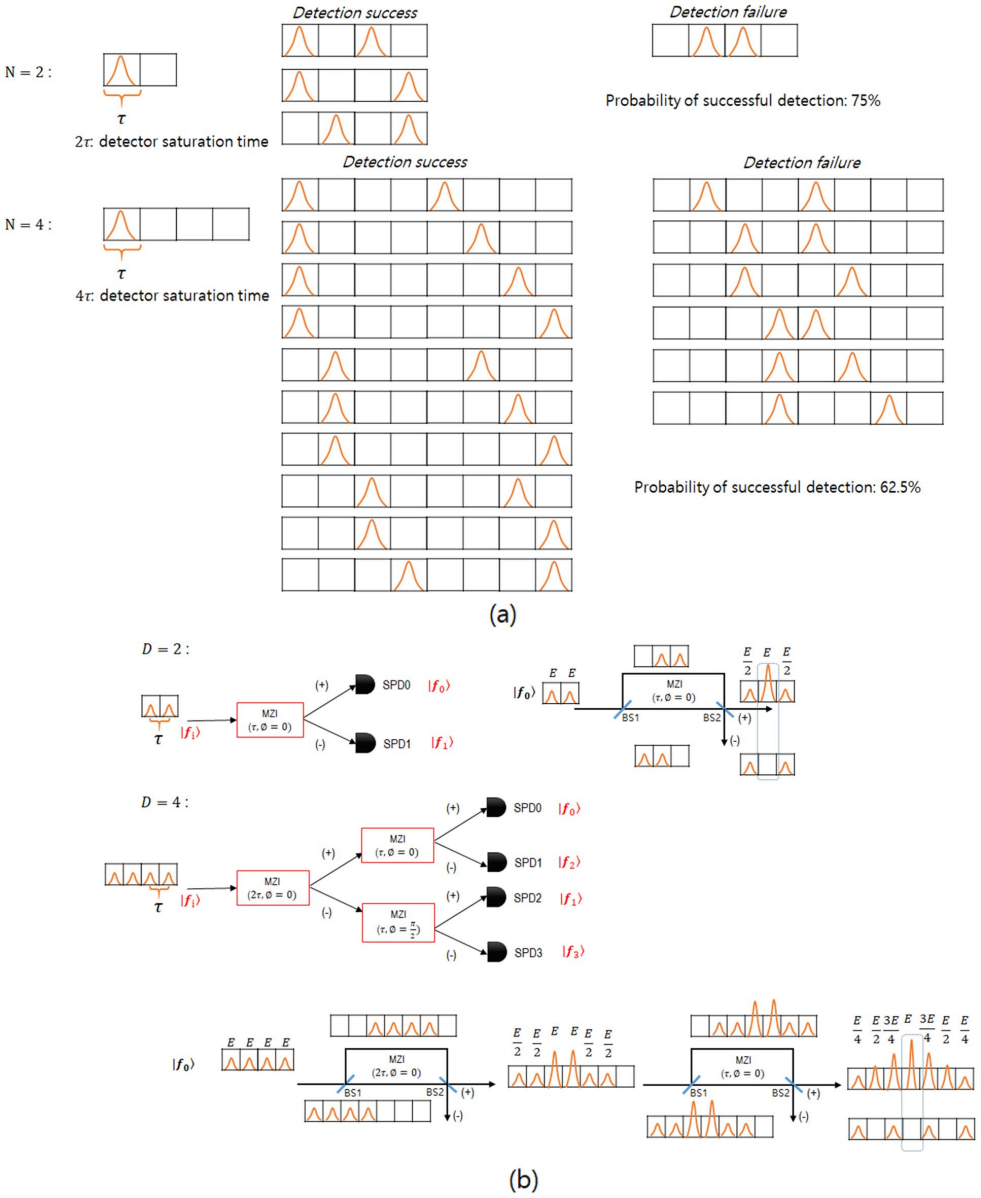
(**a**)Measurement efficiency of *N*-dimensional time state, (**b**) Measurement efficiency of *N*-dimensional phase state.

First, the structure of the *N*-dimensional initial time state $|t_{k}\rangle$ $(k = 0, \ldots ,N-1)$ is as follows. For time state, if the time interval between the two sequentially generated time states is shorter than the dead time, the SPD can not measure the time state that comes later. In other words, if the time state is generated faster than the maximum detection rate, detection efficiency is reduced, causing the loss of information transmitted. Table 1 represents the information loss during the measurement of the *N*-dimensional time states, and, as the time state dimension increases, the detection probability steadily decreases, achieving up to 50% convergence. The reason why the detection probability decreases as the dimension of the time state increases is as follows: In the *N*-dimensional time state, the dead time of the detector represents the time width occupied by the time state consisting of *N* time bins. When two *N*-dimensional time states are transmitted sequentially, detection succeeds if the time interval between which the two time states are measured is longer than the dead time of the detector. Figure 2a shows the cases in which detection succeeds and fails when the two and four dimensional time states are transmitted sequentially, respectively. When two *N*-dimensional time states are transmitted sequentially, the total number of cases that can occur is $N^2$, and the number of cases in which both time states succeed in detection is $\frac{N(N+1)}{2}$. Therefore, the probability of successful detection is $\frac{(N+1)}{2N}$.

However, if sufficient guard time is added, the information loss caused during the measurement process can be prevented.

**Table 1:** Detection probability of high-dimensional time state and phase state.

Dimension(D)	2-D (%)	4-D (%)	8-D (%)	*N*-D
Detection probability of time state	75	62.5	56.25	$\frac{100(N+1)\%}{2N}$
Detection probability of phase state	50	25	12.5	$\frac{100\%}{N}$

In QSDC protocol, *N*-dimensional encoded time state is generated with a total of $2N-1$ time bins, consisting of *N* time bins to represent *N* initial states and $N-1$ time bins to represent a $\log _{2} N$ bits message. In order for the sequentially transmitted encoded time states to be measured without loss during the detection process, the measurement interval between two consecutive time states must be longer than *N* time bins, which corresponds to the dead time of the detector. However, if an encoded time state consisting of $2N-1$ time bins is sent to the detector sequentially, it cannot always be guaranteed that the measurement interval between the preceding and following states is longer than the interval corresponding to the dead time. In the time state, a single photon exists in one of the $2N-1$ time bins and a measurement is made at that location, so if the types of time states received sequentially are different, there may be a problem that the later time state cannot be measured because the time interval between the two states where the single photon exists is shorter than the interval corresponding to the dead time. Therefore, we add a time interval corresponding to $N-1$ time bins as guard time to the time state consisting of $2N-1$ time bins, so that when the sequentially encoded time state is measured at the detector, the latter time state can be measured regardless of the type of the preceding time state. In conclusion, guard time is the minimum time interval required to ensure that the next time state is at least as far apart from the previous time state as the detector’s dead time, regardless of the type of time state previously sent to the detector. This allows us to use the time state for both purposes, detecting eavesdropping and generating encoded states through message encoding.

**Figure 3:**
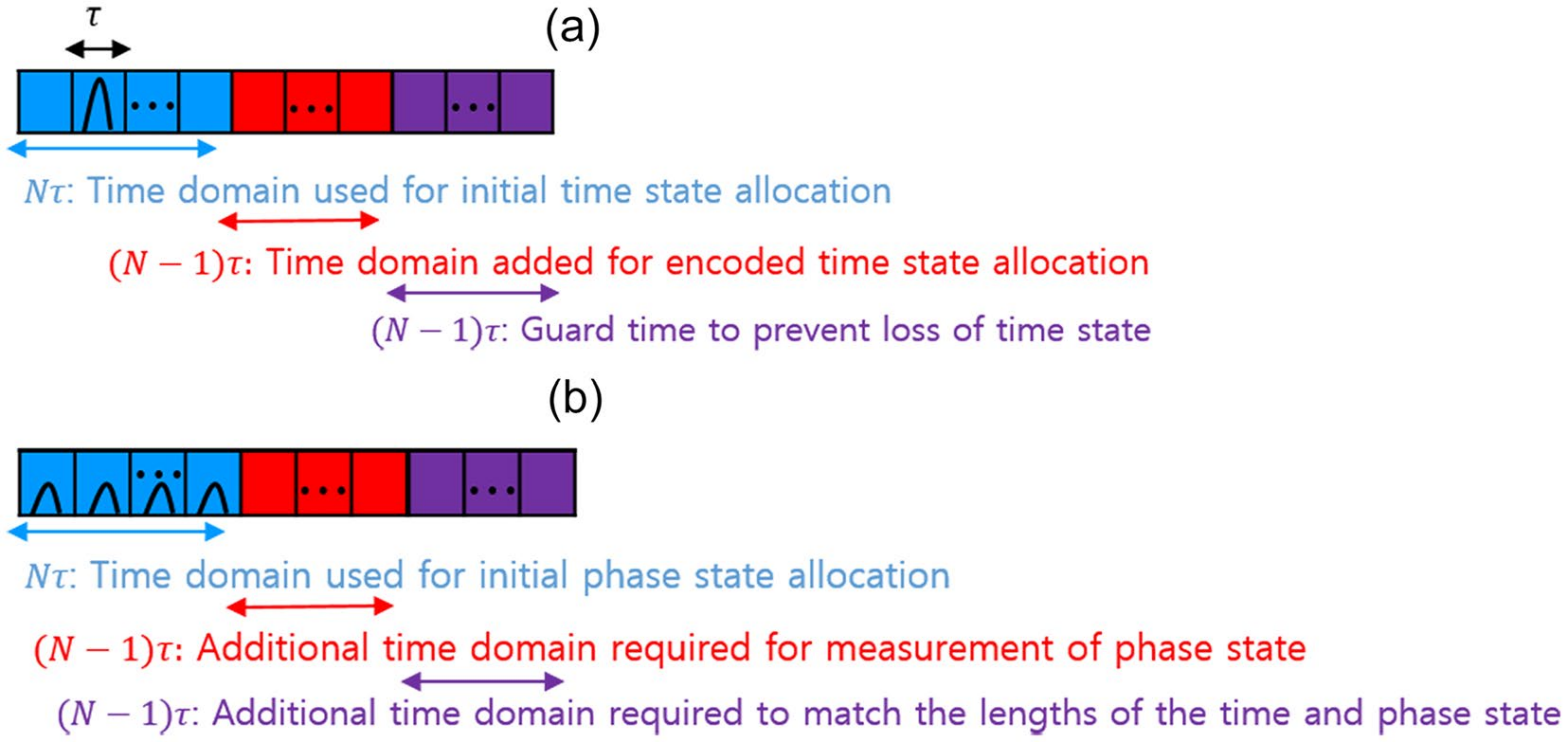
(**a**) Diagram of N-dimensional initial time state. (**b**) Diagram of N-dimensional initial phase state.

As shown in Fig. 3a, $|t_{k}\rangle$ can be expressed as photonic wave packet that has a width of $\delta$ within the $k^{th}$ time bin in a state consisting of $3N-2$ time bins, each having a width of $\tau$. Here, the reason for using the $3N-2$ time bins for forming the initial time state is that $2\textit{N}-1$ time bins are required to generate all forms of the encoded time state. To prevent the latter state from not being detected in SPD among the encoded time bin states that were sequentially sent due to dead time, $N\tau$, the guard time corresponding to the $N-1$ time bins needs to be added.

Among the two types of states applied in this study, the phase state is used only for checking the existence of the eavesdropper because detection probability is reduced by one-half as the dimension of the phase state increases two folds as shown in Table 1. The causes of the decrease in detection efficiency due to the increase in the dimension of the phase state are as follows. Since the phase state is measured using a time delay interferometer, the detection efficiency is reduced. As can be seen from the two-dimensional phase state measurement results in Fig. 2b, detection is performed in the path where constructive interference occurs in the central time bin after passing through the interferometer. At this time, the proportion of the electric field amplitude *E* of the event in the path where constructive interference occurs is half of the total, so the detection efficiency is 50%. If this is expanded to a four-dimensional phase state, as shown in Fig. 2b, the detection efficiency is further reduced to 25% because constructive output occurs at a rate of $\frac{1}{4}$ of total input intensity as it passes through the two time delay interferometers. Therefore, the detection efficiency of the *N*-dimensional phase state, which applies a detection method with a tree structure interferometric setup, is reduced to $\frac{1}{N}$. For this reason, we do not use a phase state for sending messages because the information loss becomes much greater as the dimension increases. $|p_{k}\rangle$ takes the form of a multi-peaked spectra as shown in Fig. 3b, and each peaked spectrum has a different phase value defined by the Eq. (5). $|p_{k}\rangle$ is formed by a state that has a length of $(3\textit{N}-2)\tau$ identical to the time state. Specifically, the $N\tau$ region is used for the allocation of the initial phase state, and the region amounting to $(\textit{N}-1)\tau$ is needed for measuring the phase state in a cascaded interferometric tree. The remaining $(N-1)\tau$ region is used to ensure that the total length of time and phase states are identical.

Then, Bob sends the initial states to Alice over the forward quantum channel.

#### Eavesdropping checking and measurement

After Alice receives the initial state randomly generated from Bob, QBER estimation is performed to identify the presence of an eavesdropper in the forward quantum channel using some initial states. In QBER estimation, both the initial time state and phase state are used. The beam splitter(BS) is used for random selection whether the incoming initial state is detected in the time measurement part or the phase measurement part. Here, Alice sends the measurement results, bases, and measurement positions of the initial states used in QBER estimation to Bob using public channels. Bob compares his initial information and the results received from Alice, estimates the QBER, and sends Alice the results.

**Figure 4:**
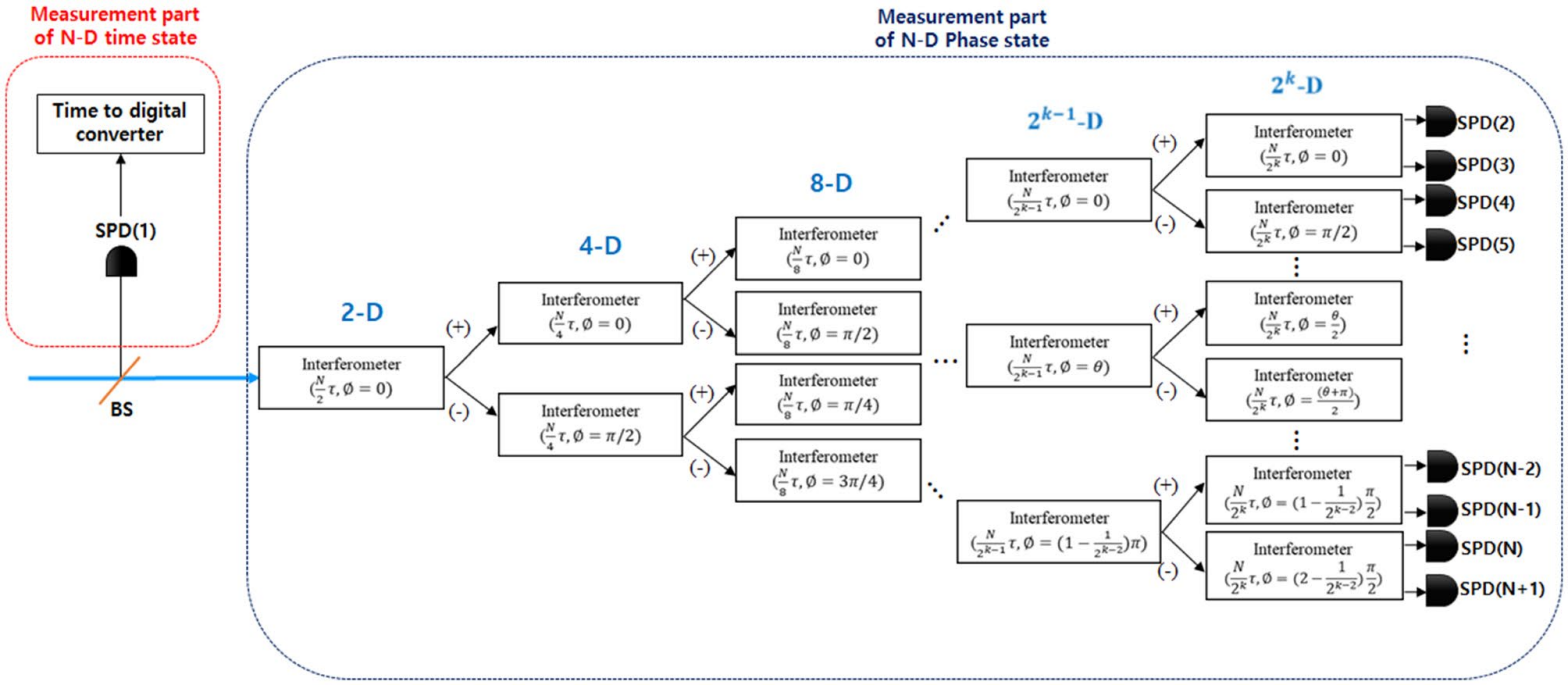
The detailed diagram of *N*(=$2^{k}$)-dimensional time and phase state measurement part.

Figure 4 shows the measurement part of the *N*-dimensional time and phase state. This part corresponds to the detection part of Alice in Fig. 1. The initial time state can connect the time-to-digital-converter with SPD (1) regardless of the dimension of the time state and measure the received time state based on the time information at which a detection event occurred in the SPD (1). For the phase state, measurement result can be obtained through the cascaded interferometric tree, which consists of $N-1$ number of time delay interferometers and *N* number of SPDs from SPD (2) to SPD ($N+1$).

**Figure 5:**
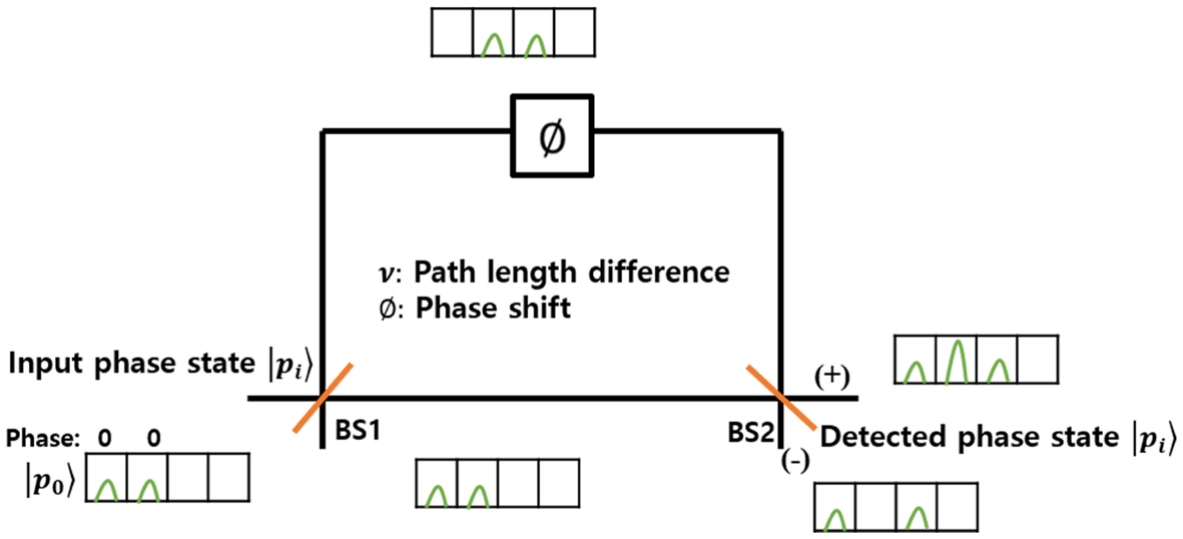
The 2-*D*(*N*=2) time delay Mach Zehnder interferometer(MZI).

The time delay Mach–Zehnder interferometers that constitute the cascaded interferometric tree consist of short and long paths with a length difference $\nu$ as shown in Fig. 5, and the phase shift $\emptyset$ is performed on the long path.

The input phase state incident to the interferometer can be measured at the location where constructive interference occurs. It is divided into two parts due to BS1 and recombined in BS2 to be detected in one of the two output paths. In the *N*-dimensional phase state, the constructive interference is observed at the $N{\text {th}}$ time bin among the $3N-2$ time bins. Among the *N* number of output paths of the cascaded interferometric tree, the constructive interference only occurs in one output path, whereas the remaining $N-1$ output paths exhibit destructive interferences at the $N{\text {th}}$ time bin. As the dimension of the phase state is increased twofold, the number of interferometers that the input phase state must pass through for measurement is increased by one. The phase shift and path length difference, which must be applied by $N-1$ number of time delay interferometers used in the cascaded interferometric tree for measuring the *N*-dimensional phase states, have the values shown in Fig. 4.

#### Storage of initial state and transmission of location information of initial time states

The initial states not used for QBER estimation are stored in the quantum memory during the QBER estimation. Alice receives from Bob the location information of the initial time state used for the message coding step via the public channel. Based on this information, we exclude the phase state, and only send the message using the time states that do not have any information loss in the measurement process.

**Figure 6:**
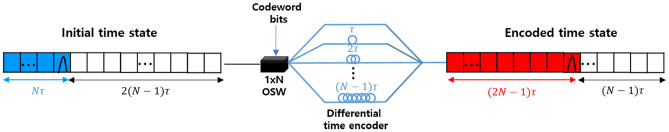
Generator of *N*-dimensional encoded time state.

#### *N*-dimensional differential time encoding

Alice generates the encoded time state that combined the codeword bits into the *N*-dimensional initial time state. Figure 6 shows the generation process of the *N*-dimensional encoded time state; we generate an encoded time state without the measurement of the initial time state using an encoding method that includes the differential time delay in the initial time state depending on the value of message bits, i.e., differential time coding, to include the message bits in the unknown N-dimensional initial time state. Table 2 illustrates the mapping rule for applying the time shift corresponding to the $\lfloor \log _{2} N \rfloor$ codeword bits to initial time state to generate the *N*-dimensional encoded time state.

**Table 2:** The mapping rule for encoded state.

$\lfloor \log _{2} N \rfloor$ Codeword bits	Length of delay time
00...$\ldots$00	0
00...$\ldots$01	$\tau$
$\vdots$	$\vdots$
11...$\ldots$11	$(N-1)\tau$

For *N*-dimensional differential time coding, we use a multi-path that has *N* number of different path lengths. Each path has a length of 0 to $(N-1)\tau$, and has a length difference of $\tau$, corresponding to the time bin width. The path, which is connected according to the value of the codeword bits allocated to the *N*-dimensional initial time state, is determined by the 1:*N* optical switch. As shown in Fig. 6, when the initial time state is converted to the encoded time state, the codeword bits are expressed as one of the photonic wave packets among the $(2N-1)^{\text {th}}$ time bins.

The encoded time state is sent to Bob through the backward quantum channel. Here, even if the encoded state is eavesdropped, The eavesdropper does not know the initial time state; thus, the message cannot be recovered from the encoded state in which the initial state and message are combined, guaranteeing the security of the message.

#### Demodulation and message recovery

Bob measures the received state. The measurement process is the same as in Fig. 4, except that we use OSW instead of BS to determine the measurement paths of the time state and phase state. Because the received state consists of a basis identical to the initial state, Bob uses the 1:2 optical switch to always detect from the measurement part having the same basis as the initial state. If the time state comes to the Bob side, the state is measured using TDC, and if the received state is the phase state, the state is measured using the cascaded interferometric tree, hence there is no information loss due to a mismatch of the measurement basis. Among the measured received states, certain parts are used for QBER estimation of the backward quantum channel.

The next step is to recover the message bits using the measurement result of the received state. By comparing the measurement values of the initial time state and encoded time state, Bob can obtain the codeword bits. For example, if the initial time state generated by Bob in the four-dimensional QSDC is $|t_{0}\rangle$, and Alice generates an encoded time state $|t_{2}\rangle$ that includes the delay time pertaining to the two time bins in $|t_{0}\rangle$ to send 2 bits information (e.g., 10), then Bob can measure $|t_{2}\rangle$. Subsequently, Bob compares the measurement results of the encoded states and initial state to identify whether the position difference between the time bins including the photonic wave packet corresponds to the two time bins, and the information received from Alice can recover the value, 10. Then, the message can be recovered through decoding after obtaining the codeword.

### Security analysis

#### Secrecy capacity analysis

In this section, we describe the process of obtaining the security capacity of *N*-dimensional QSDC protocol by applying the wiretap channel model^47,50^ used in information theoretic secure communication techniques. In the Wyner’s wiretap channel theory, the main channel is applied to message transmission between Alice and Bob, and the effects of eavesdropper and environmental noises are modeled using a wiretap channel. The secure capacity^51^ of single photon based QSDC can be defined by Wiener’s wiretap channel theory, which is defined as:

6 $$\begin{aligned} C_{s}=\max \{I(A:B)-I(A:E)\}{,} \end{aligned}$$

where *I*(*A* : *B*) and *I*(*A* : *E*) denote mutual information between Alice and Bob, and Alice and Eve, respectively.

First of all, the main channel between Alice and Bob can be defined as a cascaded channel in which *N*-ary symmetric channel and erasure channel are concatenated. The capacity of the *N*-ary symmetric channel can be expressed as $\log _{2}(N)-h_{N} (e)$, where *e* means the error rate of the $\textit{N}$-ary symmetric channel or the error rate between Alice and Bob, $\textit{N}$ is the dimension of the Hilbert space, and $h_{N}$ means the Shannon entropy function of the *N*-dimensional case, as shown in Eq. (7).

7 $$\begin{aligned} h_{N}(t)=-t\log _{2}(\frac{t}{N-1})-(1-t)\log _{2}(1-t). \end{aligned}$$

In addition, the error probability of erasure channel can be expressed as $1-Q_{Bob}$. From this, the capacity of the main channel can be written as

8 $$\begin{aligned} I(A:B)=Q_{Bob}(\log _{2}(N)- h_{N}(e)){,} \end{aligned}$$

where $Q_{Bob}$ represents the total gain of signal in Bob, and can be obtained by

9 $$\begin{aligned} Q_{Bob}=Y_{0}+1-\exp {(-l_{ch1}l_{osw}\mu )}. \end{aligned}$$

It is affected by the optical switch loss $l_{osw}$, the probability of dark counts (the yield of the vacuum state ) $Y_{0}$, the mean photon number $\mu$, and the loss $l_{ch1}(=10^{-\frac{2\alpha }{10}l})$ of the forward and backward quantum channel when the channel length between Alice and Bob is *l* km and $\alpha$ is the channel losses(average 0.2 dB/km). Error rate *e* can be described as:

10 $$\begin{aligned} e=\frac{1}{Q_{Bob}}[e_{0}Y_{0}+e_d(1-\exp {(-l_{ch1}l_{osw}\mu )})]{,} \end{aligned}$$

where $e_{0}$ and $e_d$ mean the error rates of the zero photon state and the detection error rate, respectively.

Next, mutual information *I*(*A* : *E*)^47^ between Alice and Eve represents the capacity of the wiretap channel and can be defined as follows.

11 $$\begin{aligned} I(A:E)=Q_{Eve}\cdot h_{N}(e_{t}+e_{p})=Q_{Bob}\cdot g\cdot h_{N}(e_{t}+e_{p}){,} \end{aligned}$$

where $e_{t}$ and $e_{p}$ refer to the error rates of measurement using time basis and phase basis on the Alice side, respectively. *g* represents the gap between $Q_{Eve}$ and $Q_{Bob}$, and it is a factor determined by channel loss and detection efficiency of SPD, which can be expressed by:

12 $$\begin{aligned} g=\frac{Q_{Eve}}{Q_{Bob}}=10^{(l_{ch2}+l_{d}+l_{SPD})}{,} \end{aligned}$$

where $l_{SPD}$ is the loss of a superconducting nanowire single photon detector, $l_{d}$ is the loss of an optical element, and $l_{ch2}(=10^{-\frac{\alpha }{10}l})$ represents channel loss.

Using Eqs. (8) and (11), we can obtain the secrecy capacity $C_{s}$ of the *N*-dimensional QSDC protocol.

13 $$\begin{aligned} C_{s}=Q_{Bob}[\log _{2}(N)-h_{N}(e)-g\cdot h_{N}(e_{t}+e_{p})]. \end{aligned}$$

**Figure 7:**
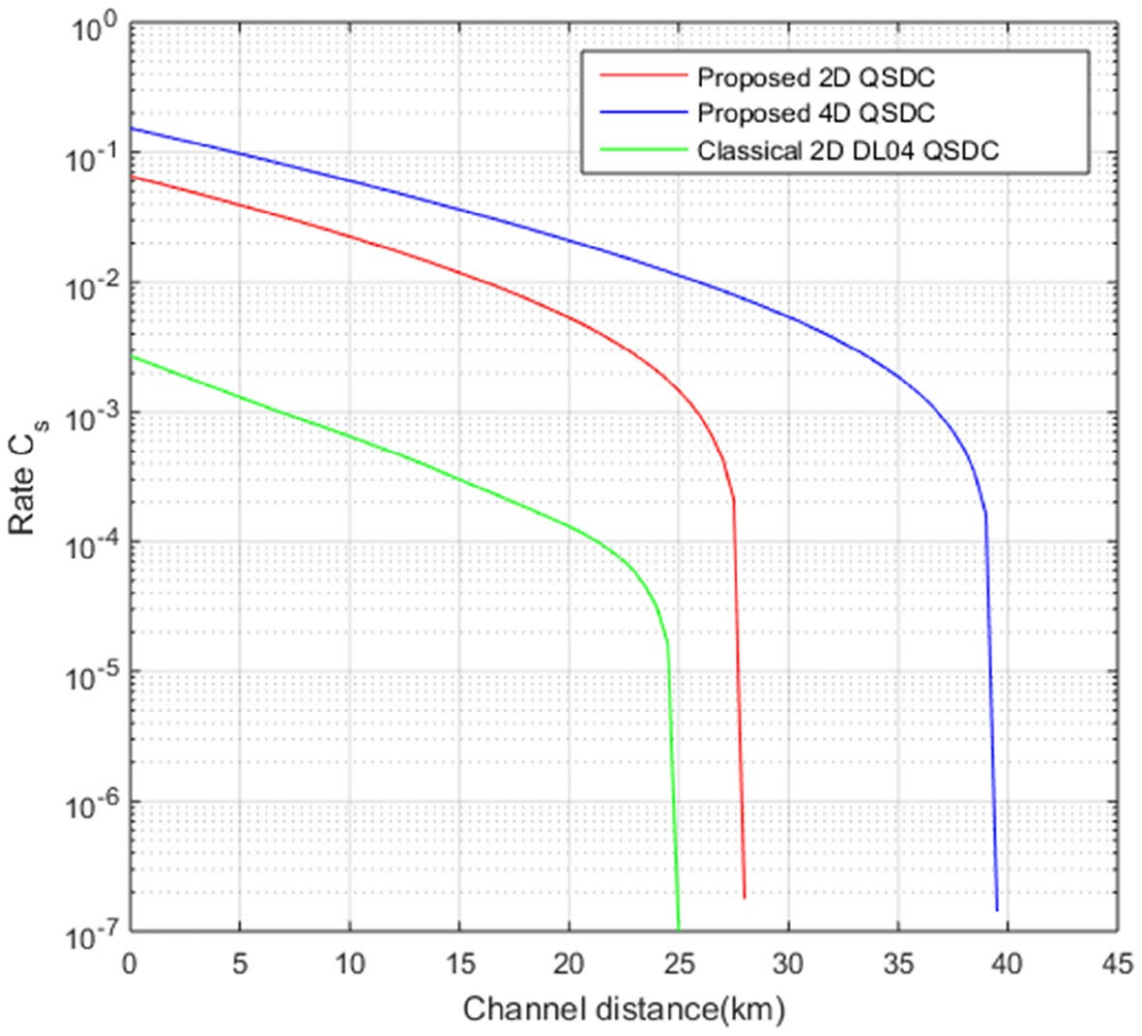
Secrecy capacity comparison between our *N*-dimensional QSDC and previous 2-dimensional QSDC^47^. We use parameters $l_{osw}$=2 dB, $Y_0=10^{-7}$, $e_d=3\times 10^{-5}$, $\mu =0.1$, $e_{t}=0.017$, $e_{p}=0.03$, $l_{SPD}=1.3dB$, $l_{d}=2.5dB$.

From Eq. (13), we can obtain the security capacity of the proposed QSDC scheme according to the channel length and dimension. As can be seen in Fig. 7, compared to the conventional two dimensional DL04 QSDC, our proposal can ensure a higher secrecy capacity and perform secure communication over a longer distance as the dimension of transmission information is higher. In addition, our scheme has improved secrecy capacity under the same channel loss conditions compared to conventional 2-dimensional scheme. In other words, conventional two-dimensional scheme can secure communication up to the maximum channel length of about 25 km, while our two and four-dimensional schemes are capable of secure and reliable communication from collective attacks up to 28 km and 40km, respectively.

#### Security of QBER estimation under intercept and resend attack

In this section, we verify the security of the proposed *N*-dimensional QSDC protocol in situation where intercept and resend attack occurs. In the intercept and resend attack, when a transmitter sends a photonic quantum state to a receiver through a quantum channel, the eavesdropper intercepts the quantum state and retransmits the quantum state corresponding to the result obtained through measurement using a random basis to the receiver.

First of all, we show that security can be guaranteed by generating high QBER when there is an intercept and resend attack by eavesdropper in the two dimensional-QSDC protocol. When the eavesdropper intercepts and measures the quantum state sent by the transmitter, the probability of using the same basis as the transmitter is $\frac{1}{2}$. In this case, the eavesdropper can always accurately measure the quantum state sent by the transmitter. However, if the basis they used does not match, half of the results measured by eavesdropper have different values from the information generated by transmitter. Based on the measurement results, the eavesdropper generates a new quantum state and sends it to the receiver. When the new quantum state arrives at the receiver, the receiver randomly selects one of the time basis and phase basis measurement methods. Receiver can be measured using the same basis as transmitter with a probability of $\frac{1}{2}$. However, due to the presence of eavesdropper, wrong results occur in an average of 25$\%$ of all events in which transmitter and receiver select the same basis, and the presence of eavesdropper can be determined from this high QBER.

Next, we verify the security in the case of extensions to *N*-dimensional QSDC protocol. Assuming the presence of the eavesdropper, in half of all events where transmitter and receiver used the same basis, the eavesdropper also uses the same basis to measure, in which case no error is generated during QBER estimation. However, if the eavesdropper uses a different basis, we can obtain wrong results with a probability of $\frac{N-1}{2N}$. This is obtained because the time basis state used in the proposed *N*-dimensional QSDC protocol is mutually unbiased with the phase basis state. If the two bases are mutually unbiased, when one of the prepared basis states is transmitted and measured on the other basis, all possible measurement results can be obtained with an equal probability of $\frac{1}{N}$. That is, considering the case where the basis of the measured basis state and the prepared basis state are different, the measured state can be measured with the same result as the prepared basis state with a probability of $\frac{1}{2N}$, but it is measured with a different result from the prepared basis state with a probability of $\frac{N-1}{2N}$. At this time, the existence of eavesdropper can be identified through QBER, which is the ratio measured as a different result.

From this, we can see that the eavesdropper generates more errors, such as 37.5$\%$ in 4-dimension and 43.7$\%$ in 8-dimensional QSDC, as the dimension of the quantum state increases. Thus, when higher-dimensional encoding techniques are applied, our QSDC protocol can ensure a higher level of security from intercept and resend attack because the transceiver can detect the presence of the eavesdropper with a higher QBER.

### Simulation results of transmission rates

Here, we investigate the improvement effect of a transmission rate of proposed QSDC with high-dimensional time and phase state compared with the classical two-dimensional QSDC.

The simulation of transmission rate considers loss of quantum channel, efficiency of SPD, storage loss of fiber-based quantum memory, dark count probability, optical switch loss, intrinsic error, and the simulation is performed based on the commercial device used in the experiment of^34,47^. Specifically, the probability of selecting the time state out of the total quantum states used in our system is $P_t(=0.5)$, the number of initial quantum states transmitted per second by Bob is $N(=6.25\times 10^{8})$, $P_c(=0.9)$ represents the ratio of quantum states used for message transmission among the total states received by Alice, and *D* is the dimension of the time state. Additionally, the detection efficiency of the superconducting nanowire SPD is $\eta _{d}=0.7$, the mean photon number $\mu$ is 0.1, the probability of dark count $Y_0$ is $10^{-7}$, and the probability of intrinsic error $e_{in}$ is set to 0.04. Since the quantum state passes through the quantum channel with length *l* twice, it can be assumed that channel loss $l_{ch}(=10^{-\frac{2\alpha }{10}l})$ occurs, and the received quantum state stored in the optical fiber based quantum memory causes storage loss while classical information is stored in the storage line during the time that classical information travels through the public channel for QBER estimation, and this loss also can be described as $l_{sl}(=10^{-\frac{2\alpha }{10}l})$.

Since only the time state is used for message transmission in our QSDC protocol using the simulation parameters defined above, the transmission rate can be estimated using the difference between the number of detection events and the number of error events in the time state. The total number of detection events in receiver’s $\textit{D}$-dimensional time state measurement part can be written as:

14 $$\begin{aligned} N_{t,D}=P_{t} P_{c} N \frac{D}{2}[1-\exp {(-l_{ch}l_{osw}l_{sl}\eta _{d}\mu )}+Y_{0}] . \end{aligned}$$

Next, the total number of error events in receiver’s *D*-dimensional time state measurement part can be represented as

15 $$\begin{aligned} E_{t,D}=P_{t} P_{c} N \frac{D}{2}[e_{in}(1-\exp {(-l_{ch}l_{osw}l_{sl}\eta _{d}\mu )})+Y_{0}]. \end{aligned}$$

From Eqs. (14) and (15), the transmission rate of the proposed *D*-dimensional QSDC can be described as

16 $$\begin{aligned} R_D=P_{t} P_{c} N \frac{D}{2} (1-e_{in})(1-\exp {(-l_{ch}l_{osw}l_{sl}\eta _{d}\mu )}). \end{aligned}$$

**Figure 8:**
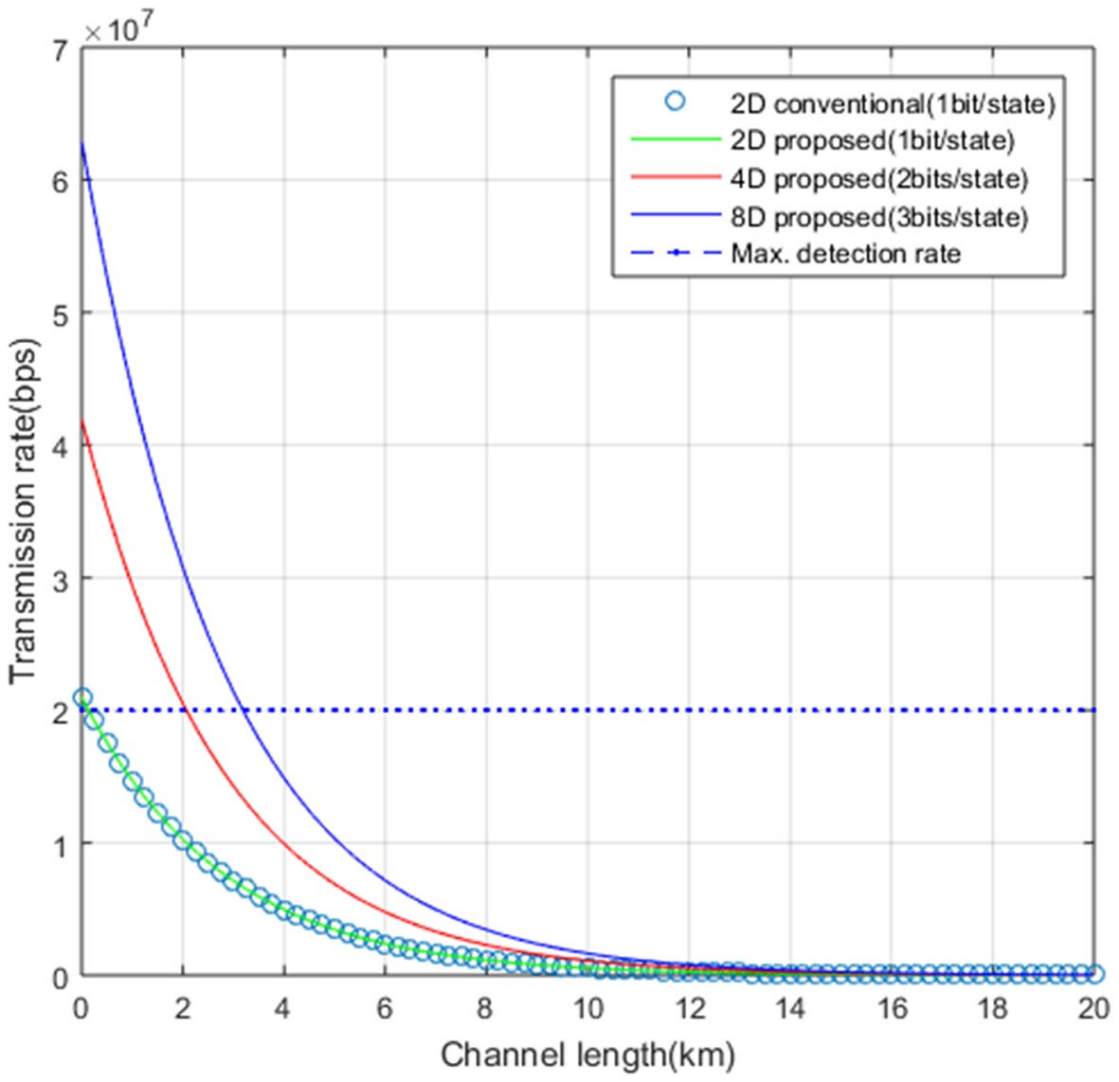
Transmission rates of our QSDC protocol according to the dimension of quantum state . Superconducting nanowire SPD with 70$\%$ efficiency and 50ns reset time is used.

In Fig. 8, we simulate transmission rates $R_D$ of *D*-dimensional QSDC with the realistic experimental factors described above. It represents the transmission rate according to the change of dimension and channel distance of the quantum state when the quantum state is generated at the same time interval in which no information loss occurs considering the dead time of SPD regardless of the dimension of the quantum state. Our scheme using higher dimensional optical quantum states has a higher transmission rate than the two-dimensional QSDC at the same channel distance, since the *D*-dimensional quantum state can carry $\lfloor \log _{2} D \rfloor$ times greater information per single quantum state compared to the 2-dimensional scheme. For example, proposed scheme can ensure a transmission rate of 1.6 Mbps in the 8D(blue solid line), 1.09 Mbps in the 4D(red solid line), and 0.57 Mbps in the 2D(green solid line) based on the same transmission distance of 10km. Due to the use of SPDs with a dead time of 50ns, no matter how fast an optical state is generated by a transmitter in a two-dimensional QSDC, a transmission rate of more than 20 Mbps cannot be obtained. However, in our 8D proposal, a transmission rate above the maximum detection rate of the detector can be obtained at a channel distance of less than 3.3km.

## Discussion

Implementation methods of the single photon based DL04 QSDC protocol using various ODOFs have been studied. In this section, we compare proposed protocol with two conventional similar protocols: One is Qi’s protocol^47^, which is based on phase state, and the other is Zhang’s protocol^46^, which implements the two-dimensional DL-04 protocol using phase and time bin state.

In Qi’s protocol, the DL-04 protocol is configured using the plug and play QKD scheme^52^. The composition method of the transceiver of the Qi’s protocol and plug and play QKD schme is the same. The initial single photon pulse prepared by Bob. Then, initial pulse is sent to Alice through the forward quantum channel. On Alice’s side, error rate checking and phase encoding are performed. Encoded state has a relative phase using a Mach–Zehnder interferometer and phase modulator. The encoded single photon state is sent to Bob in the same path as the forward channel and measured based on the relative phase difference between early and late pulses. As you can see above, the two protocols have the same configuration, but the intensity of light used in the forward quantum channel a different. The plug and play QKD protocol uses strong light, but the Qi’s QSDC scheme uses single photon. In addition, Qi’s scheme can encode one bit using a relative phase difference of two pulses in phase encoding. Therefore, it is not possible to transmit more than two bits of high-dimensional information, and this limitation allows our protocol to achieve higher transmission rates and security compared to Qi’s scheme.

In Zhang’s protocol, the DL-04 QSDC protocol is constructed by adding time bin to the classical phase-based configuration method. In an experimental setup, Bob generates two pulses using an asymmetric Mach–Zehnder interferometer and adds an intensity modulator to the phase modulator to generate time state and phase state. In this method, time bin states are applied to eavesdropping detection and are performed by measuring the time of arrival at Alice’s SPD. On the other hand, phase states are used to send a message and are measured by the result of the interference of two pulses in the asymmetric Mach–Zehnder interferometer in Bob. In the phase encoding technique using a single Mach-Zehder interferometer, as shown in Fig. 6, 1-bit classical information can be transmitted depending on the output location where constructive interference occurs. Zhang’s protocol differs from the proposed scheme in two aspects. First of all, the purpose of using time state and phase state is different. The phase state causes twice the measurement loss whenever the dimensionality of the transmitted information doubles. Therefore, because there is no effect of the high-dimensional encoding technique, the proposed scheme uses time state for message transmission, unlike Zhang’s method. In addition, our proposal can transmit multi-bit classical messages per optical quantum state, but Zhang’s scheme can only transmit one bit message. Compared with Zhang’s scheme, our technique presents a method of generating high-dimensional time and phase states, encoding messages, and measuring high-dimensional quantum states. Therefore, our protocol is more efficient than Zhang’s scheme.

In this work, we propose a high dimensional QSDC protocol that can carry more classical bits on optical quantum state generated based on two different ODOFs. Our scheme uses optical time bin and phase state, which can overcome state changes caused by scattering photon states^53^ due to turbulence occurring in wireless channels, so they are suitable for practical communication environments. We apply the *N*-dimensional time-bin encoding to the classical single-photon based QSDC to overcome the transmission rate constraint caused by the presence of detector saturation due to the dead time of SPD. Compared to the conventional two-dimensional DL04 QSDC, the proposed scheme shows that a more improved transmission rate can be obtained as the dimension *N* is gradually increased. In addition, in order to verify the security of the proposed technique in various eavesdropping situations, we first analyze it through QBER estimation according to the dimension increase of transmission quantum state, and then analyze secrecy capacity using wiretap channel in the presence of intercept and resend attack and collective attack. Through security analysis, it can be seen that our QSDC system has improved security compared to the conventional QSDC.

However, compared to DL04 QSDC, our technique increases the system complexity as the dimensionality of the quantum state increases. We compare the complexity of DL04 QSDC using time state and phase state with our protocol. First of all, in the state generation process, regardless of the dimension of the state, the same number of lasers, intensity modulators, phase modulators, and attenuators are used, so there is no difference in the system complexity of the two schemes. However, the difference in system complexity occurs during the measurement process. In the case of time state, the same number of SPDs and time to digital converters can be used for measurement regardless of the dimension of time state. The difference in complexity between DL04 and the proposed technique is due to the difference in the way phase states are measured. Since the DL04 QSDC transmits only 1 bit of information, it requires a single Mach–Zehnder interferometer and 2 SPDs to measure the phase state. On the other hand, our *N*-dimensional QSDC transmits $\lfloor \log _{2} N \rfloor$ bits of information, so it uses $N-1$ Mach–Zehnder interferometers and *N* SPDs to measure the phase state. Therefore, as the dimension of the state increases, the complexity of the measurement part increases, so a low-complexity phase state measurement method is required.

## Methods

### Basic structure: single photon based DL04 QSDC

**Figure 9:**
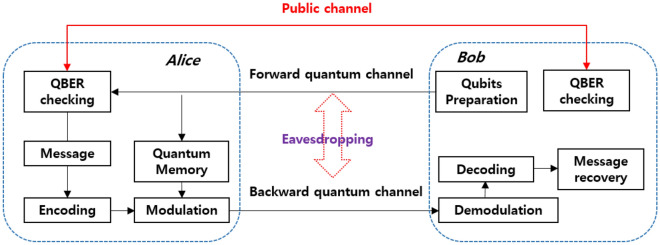
Structure of the DL04 QSDC protocol.

Our high dimensional protocol follows the process of DL04 QSDC scheme. In the DL04 protocol, the quantum state can be used to transmit information at one bit per photonic state on the two-dimensional Hilbert space. Figure 9 shows the overall architecture of the DL04 QSDC protocol.

It has the advantage of being more practical compared to the previously developed entanglement based QSDC protocol^16^. The procedure for executing the DL04 QSDC protocol is as follows.

- Bob prepares one of the four initial quantum states.
  17 $$\begin{aligned} \quad \quad \quad \quad |0\rangle =\left( \begin{array}{c} 1\\ 0\\ \end{array} \right) ,\quad |1\rangle =\left( \begin{array}{c} 0\\ 1\\ \end{array} \right) ,\quad |+\rangle =\frac{1}{\sqrt{2}}(|0\rangle +|1\rangle ),\quad |-\rangle =\frac{1}{\sqrt{2}}(|0\rangle -|1\rangle ){.} \end{aligned}$$
- Bob sends initial quantum states to Alice via the forward quantum channel.
- Alice randomly selects a part of the received initial quantum states and performs measurement using the $\textit{Z}$ and $\textit{X}$ basis. Alice then sends the selected position, basis, and measurement values to Bob using the public channel. Bob identifies the existence of the eavesdropper using the QBER estimation. If the QBER exceeds the threshold, the security of the forward quantum channel cannot be guaranteed, so the communication is stopped. If the threshold is not exceeded, the subsequent steps are proceeded.
- The initial quantum states not used in QBER estimation are saved in the quantum memory until QBER checking is complete.
- Once the forward quantum channel is secured, Alice creates the codewords by encoding the message bits. The codewords are then modulated using two types of operations. If the transmitted bit is 0, the identity operation $\textit{I}$ is executed. If the bit is 1, the unitary operation $\textit{U}$ is executed to generate encoded quantum states.
  18 $$\begin{aligned} \textit{I}=\left( \begin{array}{cc} 1 &{} 0 \\ 0 &{} 1 \\ \end{array} \right) , \textit{U}=\left( \begin{array}{cc} 0 &{} 1 \\ -1 &{} 0 \\ \end{array} \right) {.} \end{aligned}$$
  The $\textit{I}$ and $\textit{U}$ operators play a role in converting an arbitrary state into another state within the same basis. The reason why operation is performed within the same basis is that the encoded state can be accurately measured in Bob only if the basis does not change in the process of generating the encoded state in Alice.
- Alice returns the encoded quantum states to Bob through the backward quantum channel.
- Bob recovers the message information by demodulating and decoding the received quantum state.

### Experimental setup for time and phase state generation

The procedure and device configuration for generating the initial time state are shown in Fig. 1. Photonic time states are generated using a continuous-wave laser and intensity modulator. The pulse is generated using the continuous-wave laser and then modulated into desired initial time states having a desired form of narrow-width discrete optical wave packets using an intensity modulator. The intensity modulator is used for pulse shaping. Specifically, it is driven by an FPGA-based pattern generator and is responsible for defining a data pattern for each time bin that makes up the time state. By adjusting the amplitude through intensity modulation, a pulse can be present only at the position of the time bin corresponding to the data to be represented. Finally, the time state that has passed through the intensity modulator uses a variable attenuator to reduce the level of the time state to the level of a single photon.

The procedure and device configuration for generating the *N*-dimensional initial phase state are almost similar to the generation of the time state, but there is a minor difference. In the process of generating the phase state, a continuous laser and intensity modulator, as well as a phase modulator for encoding a different phase to each time bin that constitutes the phase state, are used. The phase states need to be generated through intensity modulation to ensure that the *N* number of wave packets have identical intensities, and then each wave packet is allocated with different phases using phase modulators. To create an *N*-dimensional phase state, *N* different phases must be created in the phase modulator. For example, a four-dimensional phase state uses four different phases: 0, $\frac{\pi }{2}$, $\pi$, and $\frac{3\pi }{2}$. To create the four phases, the phase modulator is driven using the four different signals obtained by the combination of the signals received from the FPGA, and the desired phase value is assigned to each time bin that constitutes the phase state. After modulation, the phase state is also passed through a variable attenuator to reduce the level of the state to the level of the single photon.

In addition, to select the initial phase and time state and base, a true random number generator is required, and all signals in the entire system are controlled by the FPGA.

### Experimental setup for time and phase state detection

The time states can be detected using a high speed time-to-digital converter connected to a single photon counting detector. The time state can be detected by using the position information of the time bin corresponding to the measured time of the single photon.

For the measurement of the *N*-dimensional phase state defined in Eq. (5), the detection method in which $N-1$ time delay Mach–Zehnder interferometers are applied in a tree structure is used as shown in Fig. 4. After constructing the multi-port version of the Mach–Zehnder interferometer as shown in Fig. 4, the detection of the optical phase state occurs at one of the output ports corresponding to being projected into one of the basis states $|p_{k}\rangle$. If we divide the arrival time of each photon by $2^{N}$ time bins, we need $2^{N}$ outcomes for every measurement that is mutually unbiased for arrival time. The rules for constructing a new row in the tree structure for measuring the N-dimension phase state are as follows. If the phase and phase difference of the interferometer in the previous row are *N*/*K* and $\theta$, respectively, the phase and phase differences of the two interferometers located in the new row connected to it are as follows, respectively. One is *N*/2*K*, $\theta /2$, and the other is *N*/2*K*, $(\theta +\pi )/2$.

### Supplementary Information

Supplementary Information.

## Data Availability

The datasets generated during and/or analysed during the current study are available from the corresponding author on reasonable request.
