# Supplementary material for: High-dimensional single photon based quantum secure direct communication using time and phase mode degrees
Source: Sci Rep. 2024 Jan 9;14:888. doi: 10.1038/s41598-024-51212-6 (PMC10776589; doi:10.1038/s41598-024-51212-6)
Supplement: Supplementary file 1 — Supplementary Information. [file 41598_2024_51212_MOESM1_ESM.pdf]

Supplementary material: High-dimensional single photon based quantum secure direct communication using time and phase mode degrees

BYUNGKYU AHN*, JOOYOUN PARK, JONGHYUN LEE, and SANGRIM LEE

Communication & Media Standard Lab., LG Electronics, Seoul, 06772, South Korea

I. Dead time of single photon detector

To detect one photon and further detect the subsequent photons, the SPD needs time interval to go back to the ready state for single photon detection. This time interval is called dead time or recovery time. The dead time varies significantly depending on the type of materials used for constituting the SPD and detector circuit design methods. Moreover, the SPD may use external electronic devices for measuring the time difference of detected photons. In this case, the SPD dead time can be determined by the dead time of external electronic devices.

Table 1 lists the key performances such as the dead time of different SPD types. The dead time limits the detection rate to saturate the transmission rate in the quantum communication. Even the superconducting nanowire single photon detector, which has been frequently used owing to its diverse advantages including a short dead time, high detection efficiency, and low dark count, is known to have a long dead time of at least 10 ns. Even though the current photon generator can generate a high-speed photonic state with short time intervals of less than 1 ps, it is difficult to expect a detection rate of 10∼100 MHz or higher due to the dead time (>10 ns) of the detector, which is 10^4 times longer than the generation interval of photonic quantum state.

	Operation Temperature(K)	Detection Efficiency(%)	Dead Time(ns)	Timing jitter(ps)
Si-SPAD [1]	263	80(800nm)	50	400
InGaAs-SPAD [2]	250	25(1550nm)	1000	150
TES [4]	0.1	90(1550nm)	<1000	100000
SNSPD [3]	2.7	85(1550nm)	10<	25

Table 1: Performances of different SPD types. (SPAD: Single photon avalanche diodes, TES: Transition edge sensor, SNSPD: Superconducting nanowire single photon detectors.)

References

[1] https://marketing.idquantique.com/acton/attachment/11868/f-0238/1/-/-/-/-/ID120Brochure.pdf.

[2] https://marketing.idquantique.com/acton/attachment/11868/f-0234/1/-/-/-/-/ID230Brochure.pdf.

[3] https://singlequantum.com/products/single-quantum-eos/.

[4] Lita, A. E., Miller, A. J., and Nam, S. W. Counting near-infrared single-photons with 95% efficiency. *Opt. Express 16*, 5 (Mar 2008), 3032–3040.
